# Transcriptomic and molecular evidence for baclofen-associated modulation of inhibitory synaptic and neuroinflammatory pathways in diabetic neuropathic pain

**DOI:** 10.3389/fphar.2026.1917799

**Published:** 2026-08-20

**Authors:** Kaisheng Ye, Qun Wang, Haishao Chen, Sizhuang Xie, Kangyu Ye

**Affiliations:** 1 Department of Anesthesiology, The First Affiliated Hospital of Jinan University, Guangzhou, Guangdong, China; 2 Department of Anesthesiology, Binhaiwan Central Hospital of Dongguan, Dongguan, Guangdong, China; 3 Department of Anesthesiology, Heyou International Hospital, Foshan, Guangdong, China

**Keywords:** baclofen, diabetic neuropathic pain, GABA_B_ receptor, GABAergic signaling, molecular docking, neuroinflammation, peripheral blood transcriptomics, whole-brain RNA-seq

## Abstract

**Background:**

Diabetic neuropathic pain (DNP) is a disabling complication of diabetes mellitus and is closely associated with impaired inhibitory neurotransmission and neuroimmune activation. Although GABAergic dysfunction has been implicated in neuropathic pain, the relationship between peripheral transcriptomic signatures, central transcriptional alterations, and the therapeutic effects of GABA receptor modulation remains incompletely understood. This study integrated human peripheral blood transcriptomic reanalysis, mouse whole-brain RNA-seq, behavioral assessment, RT-qPCR, and molecular docking to investigate DNP- associated molecular alterations and the potential mechanism of baclofen.

**Methods:**

Peripheral blood transcriptome data from diabetic peripheral neuropathy patients and healthy controls were reanalyzed using the GSE95849 dataset as a DPN/DNP-related human transcriptomic resource. Differential expression analysis, custom preranked GSEA, ssGSEA, and curated heatmap analysis were performed to identify immune-inflammatory and synaptic/GABAergic-related signatures. A streptozotocin-induced DNP mouse model was established, and DNP mice received systemic baclofen treatment. Mechanical allodynia, thermal hyperalgesia, grip strength, and motor coordination were assessed. Whole-brain RNA-seq was performed in control, DNP, and baclofen-treated DNP mice to evaluate central transcriptional alterations and treatment-associated pathway modulation. RT-qPCR was used to measure brain *Gabra1* expression, and molecular docking was performed to evaluate the predicted binding mode of baclofen with GABA receptors.

**Results:**

Reanalysis of human peripheral blood transcriptomes revealed enhanced immune-inflammatory activation and reduced synaptic/GABAergic-related pathway activity in the DPN/DNP-related human peripheral blood dataset. In DNP mice, baclofen treatment significantly alleviated mechanical allodynia and thermal hyperalgesia and partially improved neuromuscular performance without impairing motor coordination. Whole-brain RNA-seq showed that DNP mice exhibited significant negative enrichment of GABAergic signaling and inhibitory synapse signatures, together with positive enrichment of neuroinflammation/NF- *κ*B and glial activation-related signatures. Baclofen treatment showed partial and directional modulation of these DNP-associated transcriptional alterations, including a shift toward restoration of inhibitory/synaptic signatures and attenuation of neuroinflammatory pathway activity. RT-qPCR further showed that baclofen increased the DNP-suppressed brain expression of *Gabra1*. Molecular docking supported the preferential interaction of baclofen with the GABA_B_ receptor, including a predicted salt bridge with Arg162 within the Venus Flytrap domain.

**Conclusion:**

DNP is associated with convergent peripheral and central transcriptomic alterations characterized by immune-inflammatory activation and reduced inhibitory/synaptic pathway activity. Baclofen alleviates neuropathic hypersensitivity and is associated with partial, pathway-level modulation of DNP-related brain transcriptional changes, with *Gabra1* restoration and GABA_B_ receptor binding providing additional mechanistic support. These findings suggest that GABA_B_ receptor modulation may contribute to restoring inhibitory balance and attenuating neuroinflammatory activation in DNP.

## Graphical Abstract

**Figure:**
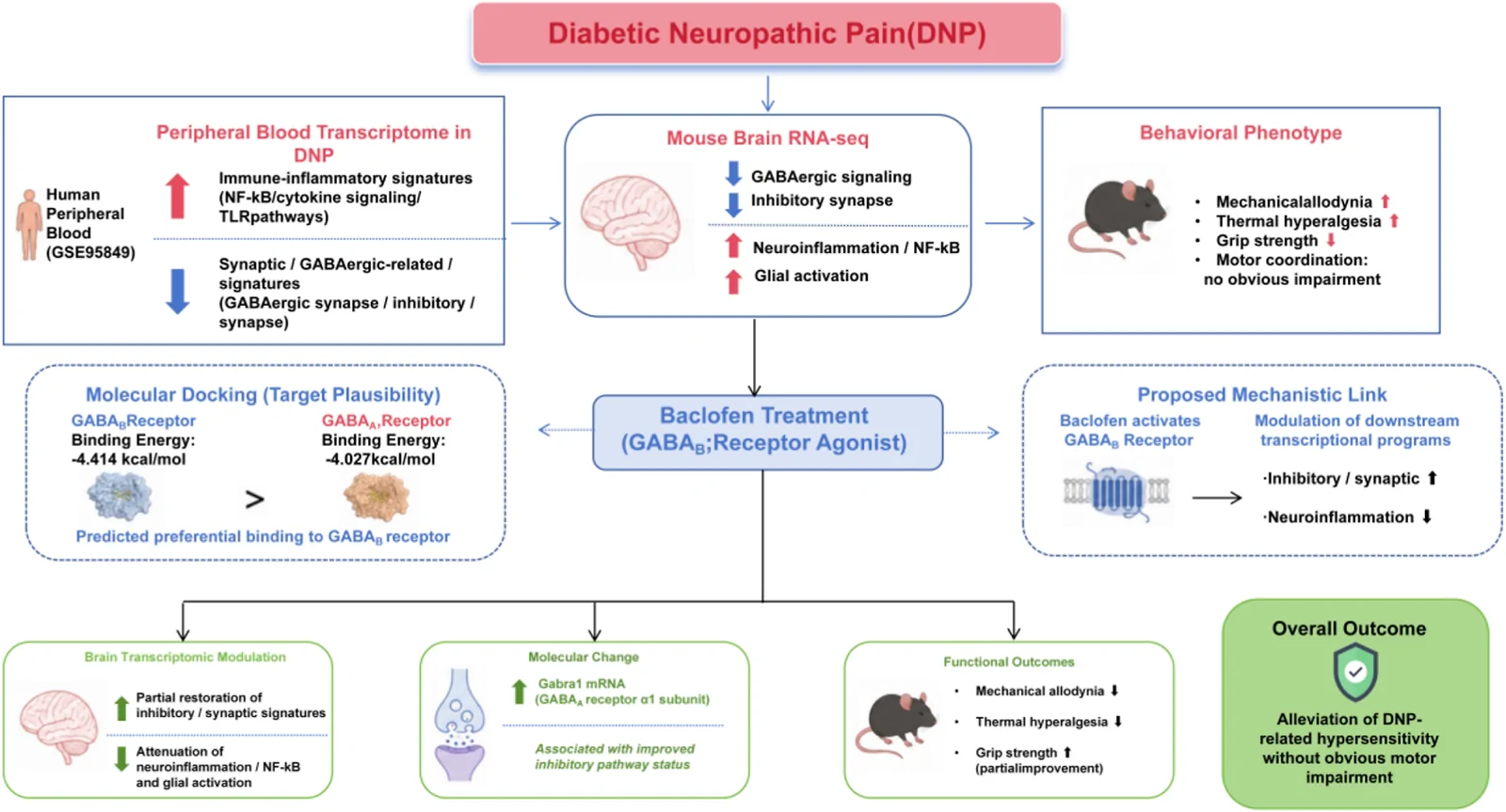

## 1 Introduction

Diabetes mellitus is a major global health burden, and its prevalence continues to rise worldwide. Among the chronic complications of diabetes, diabetic peripheral neuropathy (DPN) is one of the most frequent and disabling, affecting up to half of individuals with diabetes during the disease course (Sun et al., 2022; Feldman et al., 2019; Elafros et al., 2022). A substantial proportion of patients with DPN develop diabetic neuropathic pain (DNP), which is characterized by spontaneous pain, burning sensations, paresthesia, mechanical allodynia, and thermal hyperalgesia. These symptoms markedly impair sleep, mobility, psychological wellbeing, and quality of life, while also increasing medical costs and long-term disability (Feldman et al., 2019; Elafros et al., 2022; Savelieff et al., 2025). Current pharmacological management of painful DPN largely relies on symptomatic analgesic strategies, including gabapentinoids, serotonin–norepinephrine reuptake inhibitors, tricyclic antidepressants, sodium-channel-targeting agents, topical therapies, and neuromodulation approaches in refractory cases (Mallick-Searle and Adler, 2024; Tesfaye et al., 2022). However, many patients experience incomplete pain relief, dose-limiting adverse effects, or insufficient long-term benefit. Therefore, elucidating disease-relevant molecular mechanisms and identifying mechanism-based therapeutic strategies remain important priorities.

The pathogenesis of DNP is multifactorial and involves metabolic injury, mitochondrial dysfunction, oxidative stress, microvascular impairment, immune-inflammatory activation, peripheral sensitization, and central sensitization (Feldman et al., 2019; Elafros et al., 2022). In addition to peripheral nerve damage, increasing evidence indicates that altered excitatory–inhibitory balance within the nervous system contributes to neuropathic hypersensitivity. *γ*-Aminobutyric acid (GABA) is the principal inhibitory neurotransmitter in the mammalian central nervous system, and GABAergic inhibition plays a key role in restraining nociceptive signal amplification. Impaired GABAergic transmission, loss of inhibitory interneuron function, altered chloride homeostasis, and reduced inhibitory synaptic control have all been implicated in the development and maintenance of neuropathic pain (Li et al., 2019; Marshall et al., 2017). In painful diabetic neuropathy, spinal disinhibition has been observed in both experimental models and clinical studies, supporting the concept that defective inhibitory control contributes to pain amplification in diabetes (Marshall et al., 2017; Lee-Kubli et al., 2018).

Neuroimmune activation represents another important mechanism linking diabetes to neuropathic pain. Hyperglycemia-induced tissue injury can promote the activation of glial cells and innate immune pathways, including Toll-like receptor signaling, NF-*κ*B activation, and downstream production of pro-inflammatory cytokines. These inflammatory cascades may enhance neuronal excitability, facilitate central sensitization, and interact with inhibitory neurotransmission (Liu et al., 2018; Chen et al., 2025). Thus, DNP should not be viewed solely as a disorder of peripheral nerve degeneration, but rather as a complex neuroimmune and synaptic disorder involving reciprocal interactions between inflammatory signaling and inhibitory circuit dysfunction.

Baclofen is a selective GABA_B_ receptor agonist that has long been used clinically as an antispastic agent and has shown antinociceptive potential in neuropathic pain models. GABA_B_ receptors are metabotropic Gi/o-coupled receptors that modulate neuronal excitability by suppressing presynaptic neurotransmitter release and promoting postsynaptic hyperpolarization (Bettler and Tiao, 2006). Experimental studies have shown that activation of spinal GABA_B_ receptors can alleviate diabetic neuropathic pain and modulate pain-related signaling pathways, including NMDA receptor-associated signaling and TLR4/MyD88/NF-*κ*B inflammatory cascades (Liu et al., 2018; Liu et al., 2014). Moreover, baclofen has been reported to attenuate TLR3/TLR4-induced inflammatory signaling in glial and immune cells, suggesting that GABA_B_ receptor activation may influence both neuronal excitability and inflammatory responses (Crowley et al., 2015). Nevertheless, whether systemic baclofen treatment modulates broader DNP-associated transcriptional programs in the brain remains insufficiently understood.

Although central inhibitory dysfunction is central to neuropathic pain mechanisms, human central nervous system tissues are generally inaccessible for mechanistic transcriptomic analysis. Peripheral blood transcriptomes, in contrast, provide an accessible window into systemic immune and inflammatory states associated with disease. Importantly, GABA is not restricted to neuronal signaling; it can also regulate immune-cell activity. Human peripheral blood mononuclear cells and CD4^+^ T cells express GABA-related signaling components, and GABA has been shown to suppress cytokine release from these cells (Bhandage et al., 2018). Therefore, peripheral blood transcriptomic profiling may reveal systemic molecular correlates of DNP-related immune and inhibitory-signaling alterations. However, peripheral blood data cannot directly establish central nervous system mechanisms, and tissue-level validation in disease-relevant animal models is necessary.

In the present study, we integrated human peripheral blood transcriptomic reanalysis, mouse whole-brain RNA-seq, behavioral assessment, RT-qPCR, and molecular docking to investigate inhibitory/synaptic and neuroinflammatory mechanisms in DNP and their modulation by baclofen. First, we reanalyzed the GSE95849 peripheral blood transcriptomic dataset to identify systemic immune-inflammatory and synaptic/GABAergic-related signatures associated with DNP. Second, we established a streptozotocin-induced DNP mouse model and evaluated the effects of systemic baclofen treatment on mechanical allodynia, thermal hyperalgesia, neuromuscular performance, and motor coordination. Third, we performed exploratory whole-brain RNA-seq to assess central transcriptional alterations across control, DNP, and baclofen-treated DNP mice, with a focus on GABAergic/inhibitory signaling, synaptic transmission, neuroinflammation/NF-*κ*B, and baclofen-associated transcriptional modulation. Finally, RT-qPCR analysis of *Gabra1* and molecular docking of baclofen with GABA receptors were conducted to provide additional molecular and structural support. Through this integrated human–mouse strategy, we aimed to determine whether DNP is associated with convergent peripheral and central transcriptomic signatures and whether baclofen partially modulates DNP-associated inhibitory and inflammatory transcriptional programs.

## 2 Materials and methods

### 2.1 Human peripheral blood transcriptomic dataset and sample selection

The peripheral blood transcriptomic dataset GSE95849 was retrieved from the NCBI Gene Expression Omnibus (GEO) database (Luo et al., 2017; Barrett et al., 2013). This dataset was originally generated to profile gene expression in healthy controls, patients with diabetes mellitus, and patients with diabetic peripheral neuropathy (Luo et al., 2017). To focus on neuropathy-associated transcriptomic alterations, six healthy control samples (CN; GSM2527028–GSM2527033) and six diabetic peripheral neuropathy samples (GSM2527034–GSM2527039) were included in the present analysis. The six diabetes-only samples (GSM2527040–GSM2527045) were excluded from the main comparison. Because the original dataset was annotated as diabetic peripheral neuropathy rather than strictly painful DNP, this dataset was used to explore DPN/DNP-related peripheral blood transcriptomic signatures.

### 2.2 Preprocessing, differential expression, pathway-level enrichment, and visualization of GSE95849

The GSE95849 expression matrix and sample annotation information were downloaded and processed in R. Expression values were inspected according to their distribution and log2-transformed when appropriate. Probe annotations were mapped to official gene symbols, and probes corresponding to the same gene symbol were collapsed by retaining the probe with the highest mean expression across all included samples. The resulting gene-level expression matrix was used for downstream analysis.

Principal component analysis (PCA) was performed to evaluate the global transcriptomic separation between CN and DPN/DNP-related samples. Differential expression analysis between DPN/DNP-related and CN groups was conducted using the limma R package (Ritchie et al., 2015). Genes with an adjusted P value ≤ 0.05 and an absolute log2 fold change > 1 were considered differentially expressed. A volcano plot was generated to visualize the overall distribution of differentially expressed genes, with representative immune-inflammatory and GABAergic/inhibitory-related genes labelled.

To evaluate coordinated pathway-level changes, custom preranked gene set enrichment analysis was performed based on the GSEA framework using the fgsea package (Subramanian et al., 2005; Korotkevich et al., 2021). Genes were ranked using a signed statistic calculated as sign (log2 fold change) *×* -log10(P value). Custom gene sets related to GABAergic signaling, inhibitory synapse, synaptic transmission, neuroinflammation/NF-*κ*B, and glial/myeloid activation were analyzed. Negative normalized enrichment scores indicated enrichment toward the CN side, whereas positive normalized enrichment scores indicated enrichment toward the DPN/DNP-related side.

Official GO, KEGG, Reactome, and related gene sets were analyzed using MSigDB annotations obtained through the msigdbr package to provide supplementary pathway-level validation (Liberzon et al., 2011; Liberzon et al., 2015). Sample-level pathway activity was further estimated using the ssGSEA method implemented in the GSVA framework (Hanzelmann et al., 2013). Differences in ssGSEA scores between CN and DPN/DNP-related groups were assessed using Wilcoxon rank-sum tests followed by Benjamini–Hochberg correction.

A curated heatmap was generated using row-scaled z-scores of representative genes related to GABAergic/inhibitory signaling, synaptic transmission, and immune-inflammatory activation. All visualizations were generated in R using ggplot2, pheatmap, and related packages.

### 2.3 Experimental animals and ethical statement

To validate the bioinformatics findings in an *in vivo* system, specific pathogen-free (SPF) male C57BL/6 mice (7 weeks old, 20–22 g) were obtained from Changsheng Biotechnology Co., Ltd. Mice were housed in an SPF facility under controlled environmental conditions (23 *±* 1 °C; 12-h light/12-h dark cycle) with *ad libitum* access to standard rodent chow and water. The facility was maintained at a relative humidity of 50% *±* 10%. Bedding was changed twice weekly to ensure hygiene. All animals were allowed to acclimatize for at least 3–5 days prior to experimentation. The study protocol was approved by the Institutional Animal Care and Use Committee (IACUC) of Jinan University (Approval No. GZJLAWE-20260310–03). All procedures were conducted in strict accordance with ethical guidelines to minimize animal suffering.

### 2.4 Reagents and materials

Pharmacological agents, including Streptozotocin (STZ) and baclofen, were purchased from Sigma- Aldrich (St. Louis, MO, United States), while Tribromoethanol (Avertin) was obtained from Caisheng Biological Technology (Guangzhou, China). For molecular biology assays, all essential reagents—comprising RNAiso Blood, RNAiso Plus, the PrimeScript™ II 1st Strand cDNA Synthesis Kit, and the PrimeScript™ RT reagent Kit with gDNA Eraser—were acquired from Takara (Beijing, China). Key instrumentation employed in this study included a ViiA 7 Real-Time PCR System (Applied Biosystems, United States) for transcriptional analysis, as well as a Von Frey filament set (Ugo Basile, Italy) and a plantar test apparatus (IITC Life Science, United States) for behavioral assessments.

### 2.5 Establishment of the diabetic neuropathic pain (DNP) model

The HFD/STZ protocol was used based on previous studies showing that combined dietary metabolic stress and STZ administration can induce diabetic metabolic abnormalities and neuropathy-related sensory changes in rodents (Kou et al., 2015; Davidson et al., 2018). To establish a model of Type 2 Diabetic Neuropathic Pain (DNP), a cohort of 28 mice was initially maintained on a high-fat diet (HFD, 60% fat; Changsheng Biotechnology) for 4 weeks. Following this dietary intervention, mice were fasted daily for 6 hours (9:00 AM–3:00 PM) and administered streptozotocin (STZ; 50 mg/kg, i. p.) dissolved in citrate buffer for seven consecutive days. One week after the final injection, diabetic status was verified via fasting blood glucose (FBG) measurement; mice exhibiting FBG levels *≥* 16.7 mmol/L were deemed diabetic. To confirm the onset of neuropathy, mechanical and thermal nociceptive thresholds (PWT and PWLT) were assessed 4 weeks post-STZ administration. Only mice demonstrating both sustained hyperglycemia and significantly reduced pain thresholds compared to the Control group were identified as successful DNP models (n = 12) and enrolled in the study. Animals failing to meet these strict inclusion criteria (n = 16) were excluded.

### 2.6 Grouping and treatment

Diabetic mice were randomly assigned to the DNP model group (DNP) or the baclofen-treated DNP group (BAC) (n = 6 per group). A separate group of normal mice served as the control group (CON) (n = 6). The BAC group received intraperitoneal injections of baclofen (8 mg/kg) daily for 7 days. The CON and DNP groups received an equivalent volume of saline vehicle. Body weight and FBG levels were monitored throughout the treatment period.

### 2.7 Behavioral assessment

Behavioral tests were conducted to evaluate neuropathic pain symptoms, muscular strength, and motor coordination. All experiments were performed in a quiet environment with controlled temperature, and all apparatuses were cleaned with 70% ethanol between subjects to eliminate olfactory cues.

Mechanical hypersensitivity was assessed using von Frey filaments according to the simplified up-down method for estimating paw withdrawal thresholds (Bonin et al., 2014). Thermal hypersensitivity was assessed using the Hargreaves plantar test (Hargreaves et al., 1988). For the measurement of the Mechanical Withdrawal Threshold (PWT), mice were acclimatized in individual plexiglass chambers on a wire mesh floor for 15 min. Testing was conducted using Von Frey filaments (Ugo Basile, Italy) ranging from 0.02 g to 2.0 g following the simplified up-down method (SUDO). The 0.4 g filament was applied perpendicularly to the mid-plantar surface of the hind paw for 5–6 s; a positive response (rapid withdrawal, flinching, or licking) prompted the use of the next lighter filament, while no response led to the application of the next heavier filament. The final threshold was calculated by averaging the responses of 5 stimuli per mouse.

Subsequently, Paw Withdrawal Thermal Latency (PWLT) was measured using a plantar test apparatus (Series 8, Model 390; IITC Life Science, United States). Following a 30-min acclimatization period, a radiant heat source (30–40 mW intensity) was positioned beneath the center of the hind paw. The latency from the onset of irradiation to paw withdrawal was recorded, with a cut-off time of 20 s imposed to prevent tissue damage. Three to five trials were performed for each mouse with 5-min intervals, and the mean values were recorded.

Forelimb grip strength was measured as an index of neuromuscular performance based on previously described grip strength procedures (Meyer et al., 1979). Mice were first acclimated to the grip grid (8 cm *×* 14 cm) for 5 min 1 day prior to testing. During the formal assessment, the mouse was held by the base of the tail and lowered until its forepaws gripped the center of the grid. The animal was then pulled backward steadily along the horizontal axis until its grip was released. The maximum pull force was recorded. Each mouse underwent five trials with a 5-min inter-trial interval to ensure physical recovery. The average of the five trials was used for statistical analysis.

Motor coordination and balance were evaluated using the accelerating rotarod test, a widely used assay for motor coordination in mice (Deacon, 2013). The procedure consisted of a 3-day training phase where the rod accelerated from 4 rpm to 20 rpm over 10 s and maintained that speed for 10 min. For the formal test, the apparatus was programmed to accelerate from 4 rpm to 40 rpm over a 5-min period. The latency to fall (the time elapsed before the mouse fell from the rotating rod) was recorded. Each mouse was tested three times with a minimum inter-trial interval of 10 min, and the average latency was calculated.

All behavioral assays were performed by an investigator who was blinded to the experimental grouping of the mice to minimize observer bias.

### 2.8 Real-Time quantitative PCR (RT-qPCR)

At the conclusion of the behavioral testing period, mice were deeply anesthetized via intraperitoneal injection of 1.25% Tribromoethanol (Avertin). Brain tissues were rapidly dissected on ice, frozen in liquid nitrogen, and stored at −80 °C for subsequent molecular analysis. Total RNA was extracted from brain tissue samples using RNAiso Plus (Takara, Beijing, China) following the manufacturer’s instructions. RNA concentration and purity were determined microspectrophotometrically. Reverse transcription was performed using the PrimeScript™ II 1st Strand cDNA Synthesis Kit (Takara). Quantitative PCR was subsequently conducted using the PrimeScript™ RT reagent Kit with gDNA Eraser (Takara) on a ViiA 7 System.

The primer sequences used were as follows:

*Gabra1*: Fwd 5′-ATG​AGG​TTG​ACC​GTG​AGA​GC-3′, Rev 5′-AAA​CGT​GAC​CCA​TCT​TCT​GCT-3′ (173 bp).

*Gapdh*: Fwd 5′-ATG​TGT​CCG​TCG​TGG​ATC​TG-3′, Rev 5′-AAG​TCG​CAG​GAG​ACA​ACC​TG-3′ (142 bp).

Relative mRNA expression levels were calculated using the 2^−ΔΔCT^ method, with *Gapdh* serving as the internal control (Livak and Schmittgen, 2001).

### 2.9 Mouse whole-brain RNA-seq and transcriptomic analysis

To investigate central transcriptional alterations associated with DNP and baclofen treatment, whole-brain RNA-seq analysis was performed using brain tissues from CON, DNP, and baclofen-treated DNP mice (BAC). Three biological replicates per group were included for transcriptomic analysis. The sequencing samples corresponded to the CON group, untreated DNP model group, and baclofen-treated group. At the end of behavioral testing, mice were deeply anesthetized, and whole-brain tissues were rapidly collected, frozen in liquid nitrogen, and stored at −80 °C until RNA extraction and sequencing.

mRNA sequencing libraries were constructed and sequenced by a commercial service provider. According to the sequencing report, the libraries were generated for reference genome-based mRNA-seq analysis and were non-strand-specific. Raw sequencing data were generated in FASTQ format as paired-end reads. Raw reads were first subjected to quality assessment using FastQC v0.11.9 (Andrews, 2010) and filtered using fastp (Chen S. et al., 2018). During read filtering, adapter sequences were removed, low-quality bases with Q scores below 20 at the 3*′*end were trimmed, reads shorter than 25 bp were discarded, and ribosomal RNA reads were removed. The remaining high-quality clean reads were used for downstream analysis.

Clean reads were aligned to the mouse reference genome GRCm38.91 using HISAT2 v2.1.0 (Kim et al., 2019). Bowtie2 v2.3.4.1 was used for reference sequence alignment-related processing (Langmead and Salzberg, 2012). Gene expression was quantified using StringTie v1.3.3b (Pertea et al., 2015), and expression values were normalized using the trimmed mean of M values method. FPKM values and gene-level read counts were generated for each sample. The initial gene-level count and expression matrices generated by the sequencing service provider were used for downstream reanalysis in R.

For the present study, the gene-level expression/count matrix was further analyzed in R. Differential expression analysis of the mouse RNA-seq count matrix was performed using DESeq2 (Love et al., 2014) for three pairwise comparisons: DNP versus CON, BAC versus DNP, and BAC versus CON. For pathway-level analysis, genes were ranked using a signed statistic calculated as sign (log2 fold change) *×* −log10(P value), and preranked gene set enrichment analysis was performed using the fgsea package. Custom gene sets related to GABAergic signaling, inhibitory synapse, synaptic transmission, neuroinflammation/NF-*κ*B, and glial activation were analyzed.

Sample-level pathway activity was further estimated using single-sample gene set enrichment analysis to evaluate pathway scores across individual samples from the CON, DNP, and BAC groups. To assess baclofen-associated transcriptional modulation, a reversal analysis was performed by comparing gene-level log2 fold changes from DNP versus CON with those from BAC versus DNP. Genes showing opposite directions of change between disease induction and baclofen treatment were considered directionally modulated by baclofen. Curated heatmaps were generated using row-scaled z-scores of representative genes related to GABAergic/inhibitory signaling, synaptic transmission, neuroinflammation/glial activation, and baclofen-responsive transcriptional changes. Visualization was performed using ggplot2, pheatmap, ComplexHeatmap, and related R packages.

### 2.10 Molecular docking

To investigate the binding affinity of Baclofen for GABA receptors, molecular docking analysis was performed using crystal structures of GABA_A_ and GABA_B_ receptors retrieved from the RCSB Protein Data Bank (PDB) (Berman et al., 2000) and the two-dimensional structure of Baclofen from PubChem (Kim et al., 2023). The GABA_A_ receptor structure (PDB ID: 6CDU) and the GABA_B_ receptor structure (PDB ID: 4MQE) were selected based on previously resolved structural studies (Chen Q. et al., 2018; Geng et al., 2013). Prior to docking, receptor structures were pre-processed using PyMOL 2.4.0 to remove water molecules, co-crystallized ligands, and ions. Protein and ligand preparation was performed using AutoDockTools (Morris et al., 2009), including the addition of polar hydrogen atoms and Gasteiger charges. The ligand structure was converted to a three-dimensional (3D) format and subjected to energy minimization using ChemBio3D. Both processed receptor and ligand files were converted to PDBQT format using AutoDock Tools. The docking search space was defined by a grid box configured to fully encompass the active pockets of both receptors, and Molecular docking was conducted using AutoDock Vina (Trott and Olson, 2010). The conformation exhibiting the lowest binding energy (most negative affinity) was selected as the optimal docking pose. Finally, 3D and 2D visualization analyses of the ligand-receptor interactions were performed using PyMOL and Discovery Studio software, respectively.

### 2.11 Statistical analysis data presentation and software

All experimental data are expressed as mean *±* Standard Error of the Mean (SEM). Statistical analyses and graphical visualizations for behavioral and molecular studies were performed using GraphPad Prism software (Version 9.0, GraphPad Software, San Diego, CA, United States). Bioinformatic data processing was conducted within the R statistical environment (Version 4.4. 1).

### 2.12 Statistical tests

Comparisons between two groups were analyzed using a two-tailed unpaired Student’s t-test when appropriate. For comparisons involving three or more groups, including grip strength, rotarod performance, and *Gabra1* mRNA expression, one-way analysis of variance (ANOVA) followed by Tukey’s *post hoc* test was used. Time-course data, including body weight, fasting blood glucose, PWT, and PWLT, were analyzed using two-way repeated measures ANOVA with treatment and time as factors, followed by Tukey’s *post hoc* test. A P value ≤ 0.05 was considered statistically significant.

## 3 Results

### 3.1 Peripheral blood transcriptomic reanalysis identifies inflammatory activation and reduced synaptic/GABAergic-related signatures in a DPN/DNP-related human dataset

**FIGURE 1:**
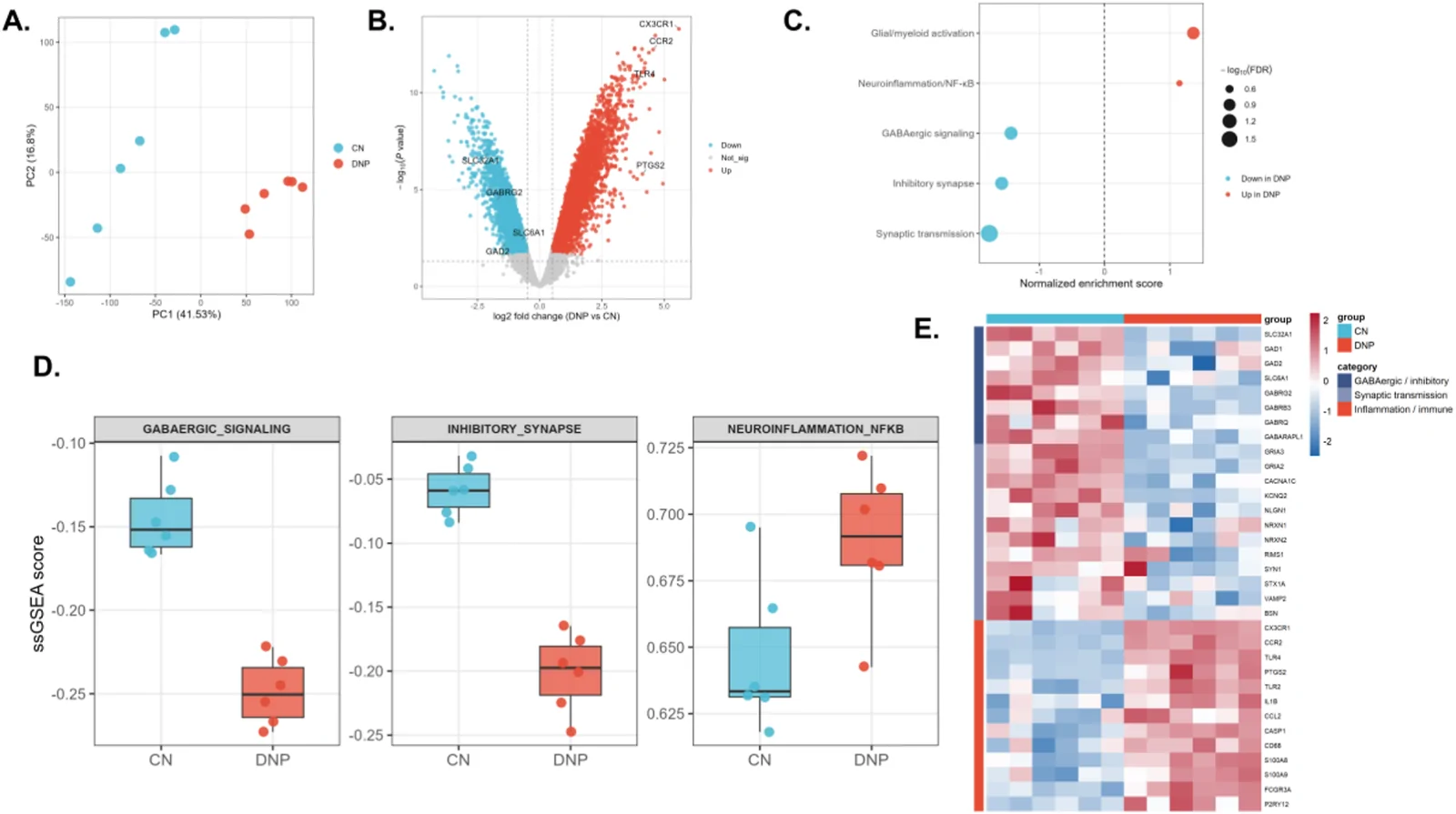
Peripheral blood transcriptomic reanalysis reveals reduced synaptic/GABAergic-related signatures and enhanced inflammatory activation in a DPN/DNP-related human dataset. **(A)** Principal component analysis (PCA) of GSE95849 peripheral blood samples from healthy controls (CN, n = 6) and diabetic peripheral neuropathy samples used as a DPN/DNP-related human transcriptomic resource (n = 6). The first two principal components explained 41.53% and 16.80% of the total variance, respectively. **(B)** Volcano plot showing differentially expressed genes between DNP and CN samples. Red dots indicate genes upregulated in DNP, blue dots indicate genes downregulated in DNP, and grey dots indicate non-significant genes. Representative immune-inflammatory genes (*CX3CR1*, *CCR2*, *TLR4*, and *PTGS2*) and GABAergic/inhibitory-related genes (*SLC32A1*, *GAD2*, *SLC6A1*, and *GABRG2*) are labelled. **(C)** Custom preranked GSEA of selected pathway signatures related to synaptic transmission, inhibitory synapse, GABAergic signaling, neuroinflammation/NF-*κ*B, and glial/myeloid activation. Negative normalized enrichment scores indicate downregulation in DNP, whereas positive normalized enrichment scores indicate upregulation in DNP. Dot size represents -log10(FDR). **(D)** Sample-level ssGSEA scores for GABAergic signaling, inhibitory synapse, and neuroinflammation/NF-*κ*B signatures in CN and DNP samples. Each dot represents one sample. **(E)** Curated heatmap of representative pathway-related genes. GABAergic/inhibitory and synaptic transmission-related genes showed lower expression in DNP samples, whereas immune-inflammatory genes showed higher expression in DNP samples. Expression values are shown as row-scaled z-scores.

To characterize peripheral blood transcriptomic alterations associated with diabetic neuropathy-related pain mechanisms, we reanalyzed the GSE95849 dataset, including six healthy control samples and six diabetic peripheral neuropathy samples used as a DPN/DNP-related human transcriptomic resource. After probe annotation, gene-level collapsing, and normalization, principal component analysis showed a clear separation tendency between CN and DNP samples, indicating distinct global transcriptomic profiles between the two groups (Figure 1A).

Differential expression analysis identified 5,200 differentially expressed genes in DNP samples compared with CN samples, including 3,653 upregulated and 1,547 downregulated genes. The volcano plot showed that several immune-inflammatory genes, including *CX3CR1, CCR2, TLR4*, and *PTGS2*, were markedly upregulated in DNP samples. In contrast, representative genes related to GABAergic/inhibitory signaling, including *SLC32A1*, *GAD2*, *SLC6A1*, and *GABRG2*, were downregulated (Figure 1B).

To determine whether these gene-level alterations reflected coordinated pathway-level changes, we performed custom preranked GSEA using selected signatures related to GABAergic signaling, inhibitory synapse, synaptic transmission, neuroinflammation/NF-*κ*B, and glial/myeloid activation. Synaptic transmission showed significant negative enrichment in DNP samples, whereas GABAergic signaling and inhibitory synapse signatures displayed consistent negative enrichment trends. In contrast, glial/myeloid activation and neuroinflammation/NF-*κ*B-related signatures showed positive enrichment trends, suggesting that DNP peripheral blood is characterized by reduced synaptic/GABAergic-related pathway activity and enhanced inflammatory activation (Figure 1C).

Consistent with the preranked GSEA results, sample-level ssGSEA analysis further demonstrated significantly lower GABAergic signaling and inhibitory synapse scores in DNP samples, together with increased neuroinflammation/NF-*κ*B pathway scores (Figure 1D). These findings indicate that the observed pathway-level alterations were also detectable at the individual-sample level.

A curated heatmap of representative pathway-related genes further supported this reciprocal expression pattern. Genes associated with GABAergic/inhibitory signaling and synaptic transmission, including *SLC32A1*, *GAD1*, *GAD2*, *SLC6A1*, *GABRG2*, *GABRB3*, *SYN1*, *STX1A*, and *VAMP2*, were generally lower in DNP samples. Conversely, immune-inflammatory genes, including *CX3CR1*, *CCR2*, *TLR4*, *PTGS2*, *TLR2*, *IL1B*, *CCL2*, *CASP1*, *CD68*, *S100A8*, and *S100A9*, were higher in DNP samples (Figure 1E). Together, these results suggest that this DPN/DNP-related human peripheral blood dataset exhibits transcriptomic signatures characterized by enhanced immune-inflammatory activation and reduced synaptic/GABAergic-related pathway activity.

### 3.2 Establishment and characterization of the diabetic neuropathic pain (DNP) model

**FIGURE 2:**
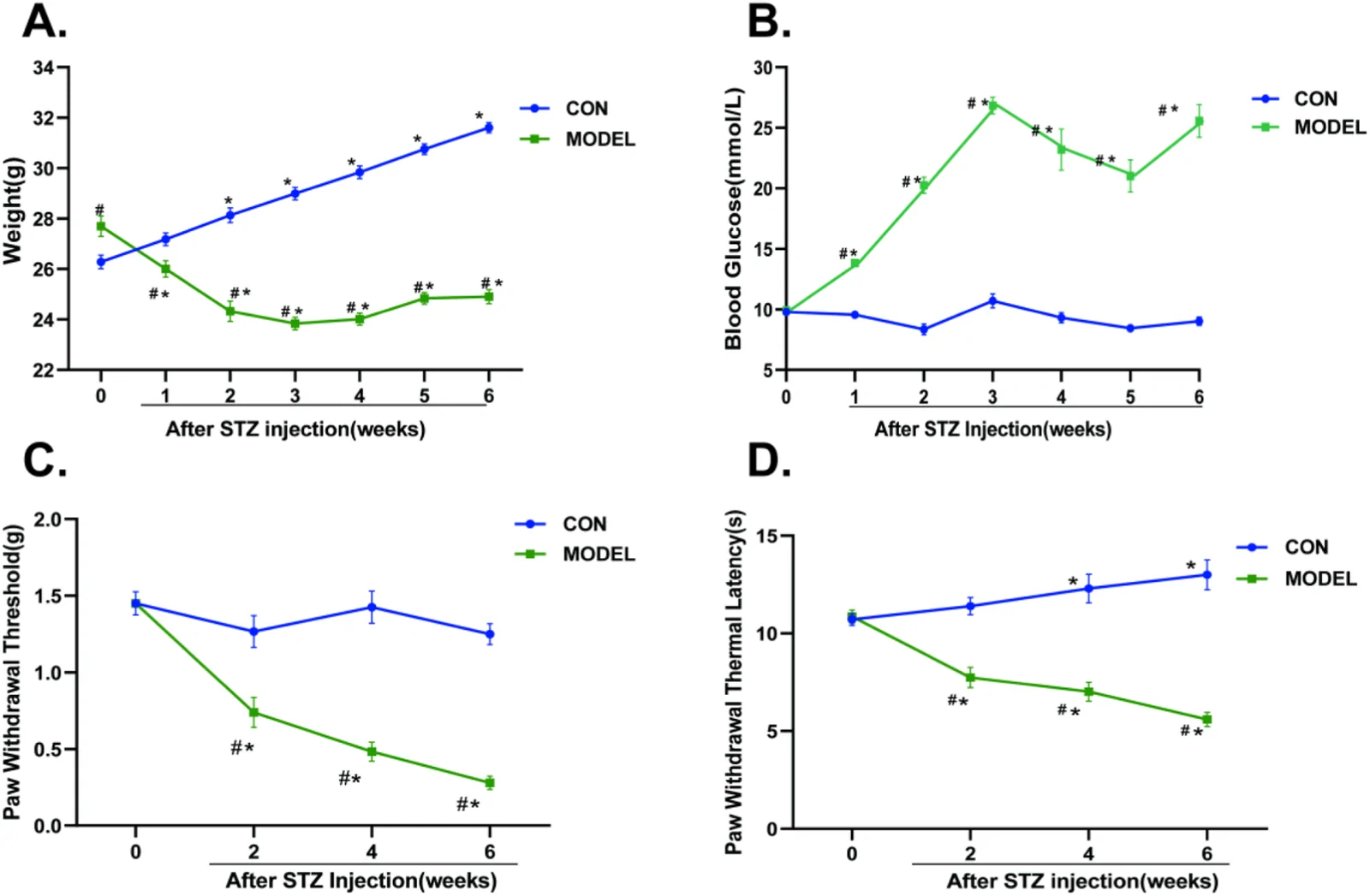
Characterization of the STZ-induced Diabetic Neuropathic Pain (DNP) model. **(A)** Time course of body weight changes in Control (CON) and DNP mice over 6 weeks. **(B)** Monitoring of fasting blood glucose levels; hyperglycemia (≥16.7 mmol/L) was sustained in DNP mice from Week 1 onwards. **(C)** Assessment of mechanical allodynia via Von Frey filaments (Paw Withdrawal Threshold, PWT). **(D)** Assessment of thermal hyperalgesia via the Hargreaves test (Paw Withdrawal Thermal Latency, PWLT). Data are presented as mean *±* SEM (n = 6 per group). Statistical significance was determined using two-way repeated measures ANOVA followed by Tukey’s *post hoc* test. #, P ≤ 0.05 vs. CON group at the same time point *, P > 0.05 vs. baseline (Week 0) within the same group.

Monitoring of metabolic parameters confirmed the successful induction of the diabetic state following STZ administration. Body weight trajectories diverged significantly between groups; while the CON group exhibited a steady weight gain throughout the study period, the DNP group displayed a progressive decline starting 1 week post-STZ injection (P < 0.05), reaching a nadir at Week 3 (23.83 *±* 0.85 g) (Figure 2A). Concurrently, blood glucose levels in the DNP group rose sharply within the first week and maintained a stable hyperglycemic state (≥ 16.7 mmol/L) from Week 2 through Week 6 (P < 0.05), peaking at Week 3 (26.8 *±* 2.4 mmol/L) (Figure 2B). In contrast, the CON group maintained normoglycemic levels throughout the experiment.

The onset of neuropathy was validated by a significant reduction in nociceptive thresholds. Prior to STZ injection, baseline Paw Withdrawal Thresholds (PWT) and Paw Withdrawal Thermal Latencies (PWLT) were indistinguishable between groups (P ≥ 0.05). However, post-induction, the DNP group exhibited a marked and sustained hypersensitivity. Mechanical thresholds (PWT) in DNP mice dropped significantly at Weeks 2, 4, and 6 compared to both baseline and age-matched controls (P < 0.05) (Figure 2C). Similarly, thermal latencies (PWLT) in the DNP group were significantly shortened at all post-induction time points (P < 0.05), confirming the development of both mechanical allodynia and thermal hyperalgesia characteristic of the DNP phenotype (Figure 2D).

### 3.3 Baclofen alleviates diabetic neuropathic pain and improves neuromuscular performance without motor impairment

Baseline assessments confirmed the establishment of neuropathic hypersensitivity before treatment. Prior to baclofen administration, both the DNP and BAC groups showed significantly reduced paw withdrawal thresholds (PWT) and paw withdrawal thermal latencies (PWLT) compared with the CON group, with no detectable difference between the two disease groups. After 7 days of treatment, baclofen significantly increased PWT and prolonged PWLT compared with untreated DNP mice, indicating attenuation of mechanical allodynia and thermal hyperalgesia. In contrast, the untreated DNP group maintained low sensory thresholds during the same period.

Motor-related behavioral tests were performed to evaluate whether the analgesic-like effects of baclofen were confounded by motor impairment. Forelimb grip strength was reduced in DNP mice compared with controls, whereas BAC-treated mice showed a significant improvement relative to untreated DNP mice, although the values did not fully return to control levels. In the accelerating rotarod test, BAC-treated mice did not show impaired motor coordination compared with controls, suggesting that the dose used in this study did not produce detectable motor suppression under the present experimental conditions.

**FIGURE 3:**
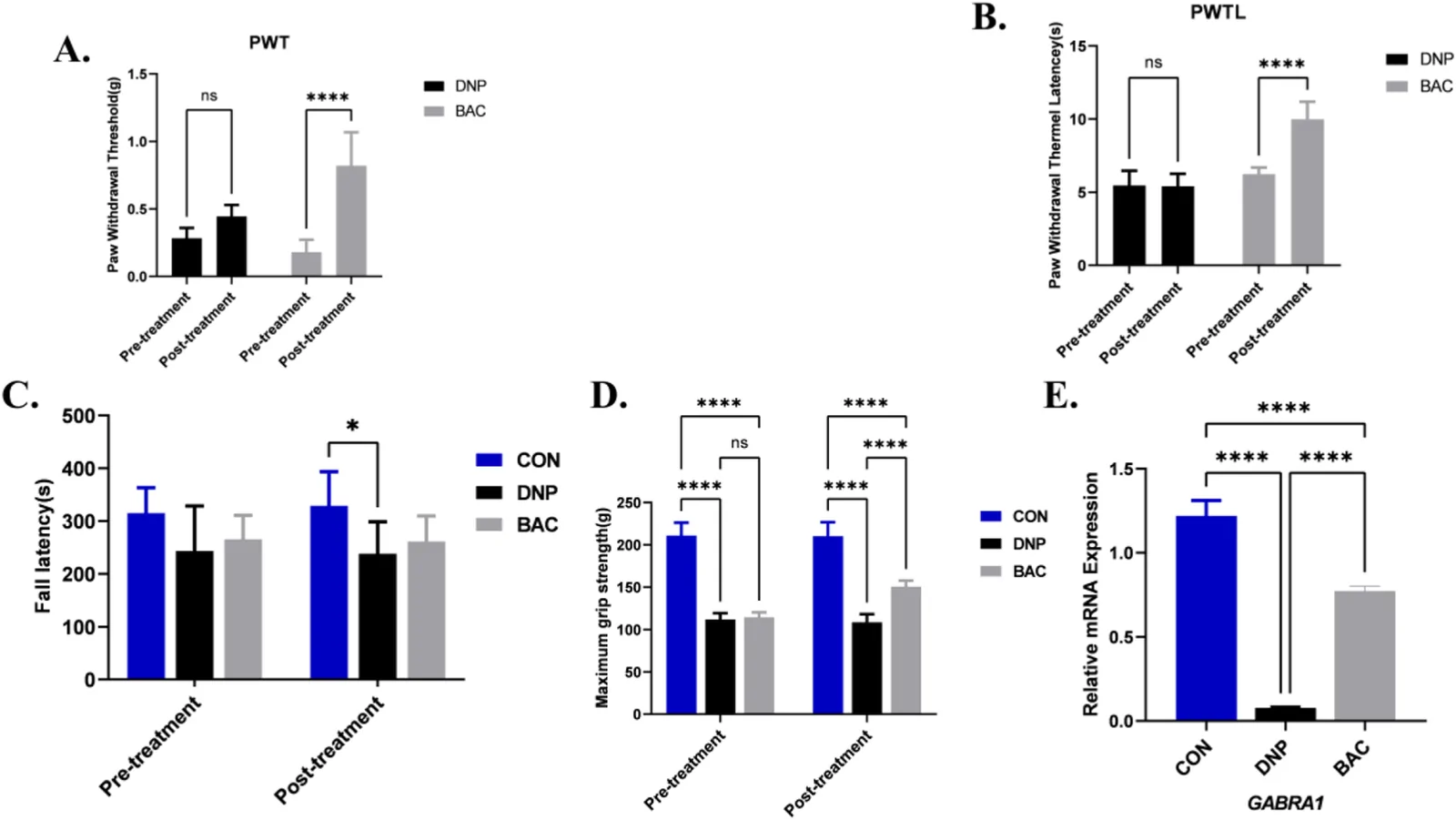
Baclofen treatment improves pain-related behaviors and neuromuscular performance without motor impairment in DNP mice. **(A)** Mechanical paw withdrawal threshold (PWT) was measured to evaluate mechanical allodynia. **(B)** Paw withdrawal thermal latency (PWLT) was measured to evaluate thermal hyperalgesia. **(C)** Latency to fall in the accelerating rotarod test was recorded as an index of motor coordination and balance. **(D)** Maximum forelimb grip strength was assessed to determine neuromuscular performance. **(E)** Relative mRNA expression levels of *Gabra1* in the brain were quantified via qPCR and normalized to internal controls. CON, normal control group; DNP, untreated diabetic neuropathic pain group; BAC, baclofen-treated DNP group. Data are presented as mean ± SEM (n = 6 per group). Statistical significance was determined by one-way or two-way ANOVA as appropriate. ns, non-significant; *P < 0.05, **P < 0.0001.

RT-qPCR analysis showed that brain *Gabra1* mRNA expression was significantly reduced in DNP mice and increased after baclofen treatment. These findings suggest that the behavioral improvement induced by baclofen was accompanied by partial restoration of an inhibitory receptor-related transcriptional marker (Figure 3).

### 3.4 Mouse brain transcriptomic analysis reveals inhibitory/synaptic suppression, neuroinflammatory activation, and partial transcriptional modulation by baclofen

**FIGURE 4:**
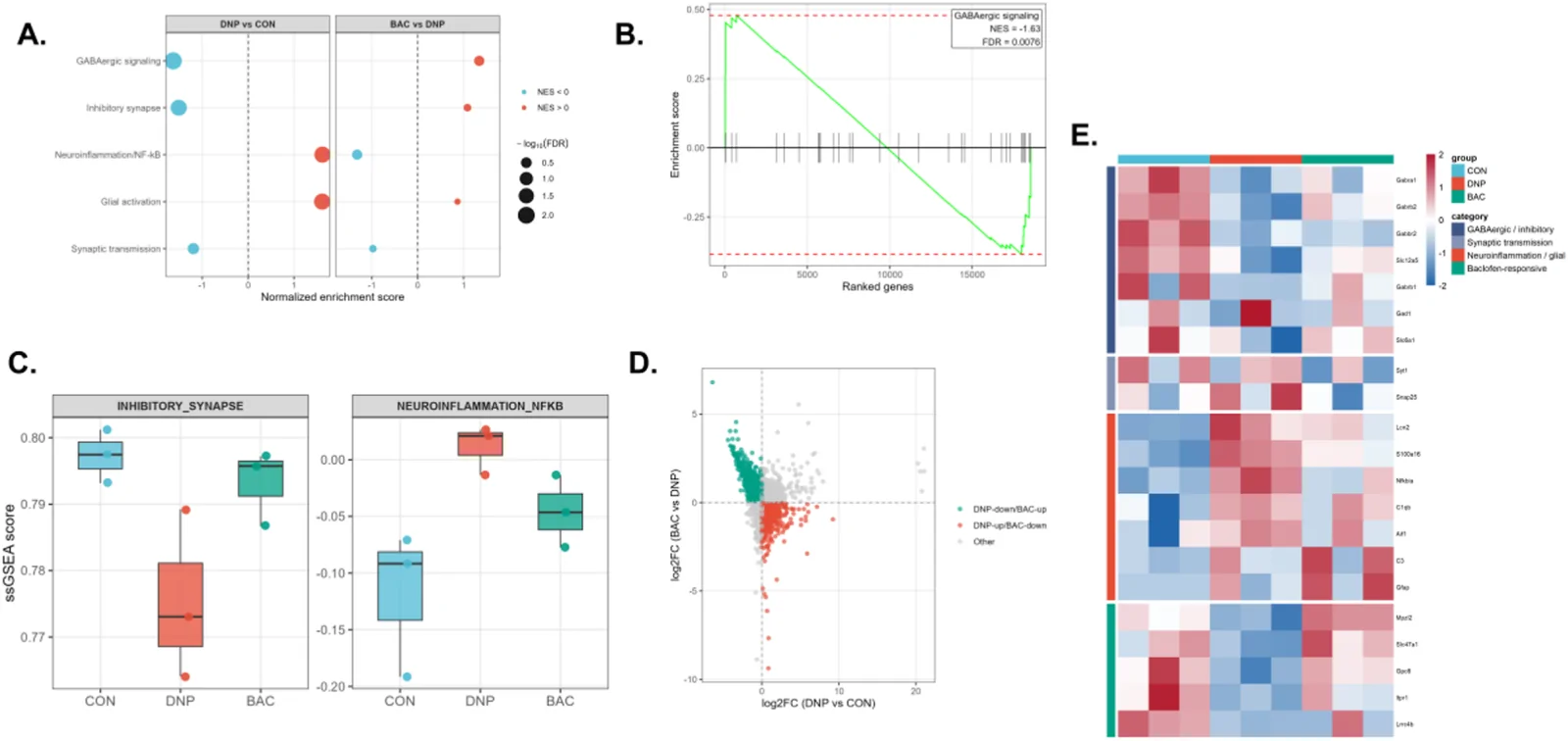
Whole-brain RNA-seq analysis reveals inhibitory/synaptic transcriptional suppression, neuroinflammatory activation, and partial baclofen-associated modulation in DNP mice. **(A)** Custom preranked GSEA dotplot showing pathway-level enrichment patterns in DNP versus CON and BAC versus DNP comparisons. Negative normalized enrichment scores indicate downregulation in the first group of each comparison, whereas positive normalized enrichment scores indicate upregulation. Dot size represents -log10(FDR). **(B)** Representative GSEA enrichment plot showing significant negative enrichment of the GABAergic signaling signature in DNP mouse brain samples compared with CON samples. The normalized enrichment score and FDR are shown within the plot. **(C)** Sample-level ssGSEA scores for inhibitory synapse and neuroinflammation/NF-*κ*B signatures across CON, DNP, and BAC groups. Each dot represents one biological sample. **(D)** Baclofen reversal quadrant plot comparing gene-level log2 fold changes from DNP versus CON with those from BAC versus DNP. Genes located in opposite-direction quadrants represent transcripts altered in DNP and directionally modulated by baclofen treatment. **(E)** Curated heatmap of representative genes related to GABAergic/inhibitory signaling, synaptic transmission, neuroinflammation/glial activation, and baclofen-responsive transcriptional changes. Expression values are shown as row-scaled z-scores. CON, control group; DNP, diabetic neuropathic pain model group; BAC, baclofen-treated DNP group.

To further explore central transcriptional programs associated with DNP and baclofen treatment, we performed exploratory whole-brain RNA-seq analysis in CON, DNP, and baclofen-treated DNP mice. Custom preranked GSEA revealed a distinct pathway-level disturbance in DNP mouse brain samples compared with CON samples. GABAergic signaling was significantly negatively enriched in DNP mice (NES = −1.63, FDR = 0.0076), and the inhibitory synapse signature also showed significant negative enrichment (NES = −1.51, FDR = 0.017). In contrast, neuroinflammation/NF-*κ*B and glial activation-related signatures were positively enriched in DNP mice (both FDR = 0.017), indicating that the DNP brain transcriptome was characterized by reduced inhibitory/synaptic pathway activity together with enhanced neuroinflammatory activation (Figure 4A).

In the BAC versus DNP comparison, baclofen treatment showed an opposite directional pattern for several DNP-associated pathways. GABAergic signaling and inhibitory synapse signatures shifted toward positive enrichment, whereas the neuroinflammation/NF-*κ*B signature shifted toward negative enrichment. Although these treatment-associated enrichments did not reach FDR significance, their directions were consistent with a partial pathway-level modulation of DNP-associated transcriptional alterations (Figure 4A). A representative GSEA curve further confirmed the significant negative enrichment of the GABAergic signaling signature in DNP versus CON, supporting suppression of GABAergic-related transcriptional programs in DNP mouse brain tissue (Figure 4B).

Sample-level ssGSEA analysis provided additional support for these pathway-level findings. Inhibitory synapse scores were reduced in the DNP group and showed a partial upward shift after baclofen treatment.

Conversely, neuroinflammation/NF-*κ*B scores were increased in DNP mice and decreased in the BAC group (Figure 4C). These findings suggest that DNP-like mice exhibit a central transcriptional profile involving impaired inhibitory/synaptic signaling and enhanced inflammatory pathway activity, while baclofen treatment is associated with partial normalization of selected pathway signatures.

To assess whether baclofen induced transcriptional changes opposite to the DNP-associated pattern, we generated a reversal quadrant plot comparing log2 fold changes from DNP versus CON with those from BAC versus DNP. A subset of transcripts was located in the opposite-direction quadrants, indicating that baclofen directionally modulated part of the DNP-associated transcriptomic alterations rather than globally restoring the entire brain transcriptome (Figure 4D). This supports a selective and partial transcriptional response to baclofen treatment.

A curated heatmap further summarized the expression patterns of representative pathway-related genes. Genes involved in GABAergic/inhibitory signaling and synaptic transmission, including GABA receptor subunits, chloride transporters, and synaptic vesicle-associated genes, tended to show lower expression in the DNP group. In contrast, neuroinflammation/glial-related genes showed higher expression in DNP mice. Baclofen-treated mice displayed partial shifts toward the CON pattern in selected inhibitory/synaptic, inflammatory, and baclofen-responsive genes (Figure 4E). Together, these whole-brain RNA-seq findings indicate that DNP-like mice exhibit inhibitory/synaptic transcriptional suppression and neuroinflammatory activation, and that baclofen partially and directionally modulates a subset of these DNP-associated brain transcriptional changes.

### 3.5 Structural basis for target selectivity

**FIGURE 5:**
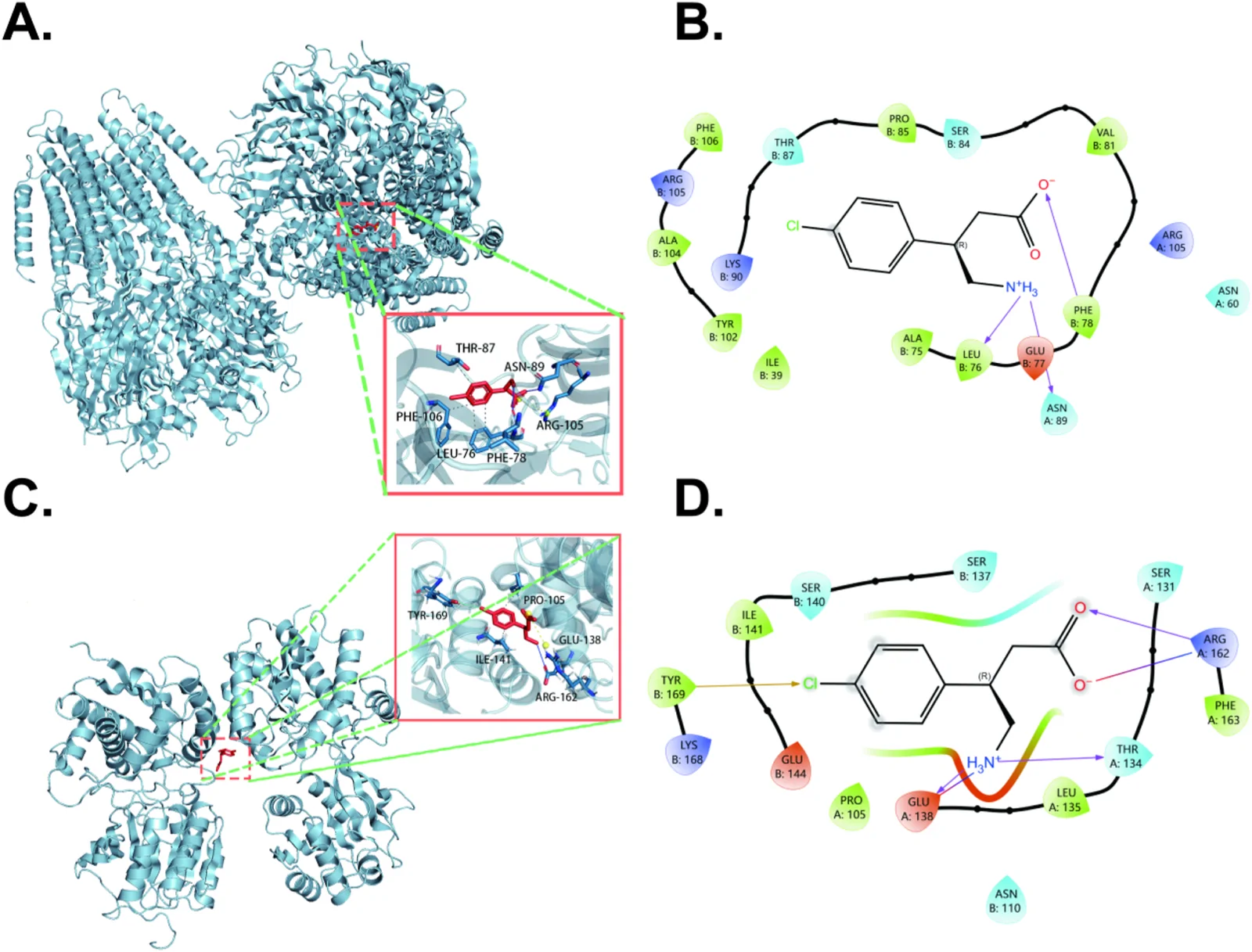
Molecular docking analysis and predicted binding conformations of baclofen with GABA_A_ and GABA_B_ receptors. **(A)** Three-dimensional binding conformation of baclofen docked into the GABA_A_ receptor complex (PDB ID: 6CDU). **(B)** Two-dimensional schematic representation of the predicted binding site of baclofen within the GABA_A_ receptor. **(C)** Three-dimensional binding conformation of baclofen docked into the GABA_B_ receptor complex (PDB ID: 4MQE). **(D)** Two-dimensional schematic representation of the predicted binding site of baclofen within the GABA_B_ receptor.

Molecular docking analysis was performed to evaluate the predicted interaction between baclofen and GABA receptors. Baclofen showed a slightly more favorable predicted binding energy with the GABA_B_ receptor (−4.414 kcal/mol) than with the GABA_A_ receptor (−4.027 kcal/mol). The predicted binding energies and key residue interactions are summarized in Table 1. Structural analysis suggested that baclofen could be accommodated within the GABA_B_ Venus Flytrap domain through polar and hydrophobic interactions, including a predicted salt bridge with ARG A:162 and a hydrogen bond with GLU A:138. In comparison, the predicted GABA_A_ binding pose involved a different interaction pattern, including hydrogen bonds with LEU 76B, PHE 78B, and ASN 89A, as well as a predicted salt bridge with ARG 105A. These docking results are consistent with the known pharmacological preference of baclofen for GABA_B_ receptors and provide structural support for target plausibility (Figure 5). However, docking analysis alone cannot establish receptor activation, binding kinetics, or downstream transcriptional effects.

**TABLE 1:** Molecular docking analysis of the predicted binding affinity and key residue interactions between baclofen and GABA receptors.

Target	PDB ID	Binding energy (kcal/mol)	Key polar interactions (residue, length A˚)	Hydrophobic & other interactions (residue, length A˚)
GABA_A_ GABA_B_	6CDU 4MQE	−4.027 −4.414	LEU 76B (H-bond, 1.81) PHE 78B (H-bond, 1.95) ASN 89A (H-bond, 2.62) ARG 105A (salt bridge, 5.33) GLU A:138 (H-bond, 1.75) ARG A:162 (salt bridge, 4.50)	PHE 78B (hydrophobic, 3.74) THR 87B (hydrophobic, 3.63) PHE 106B (hydrophobic, 3.90) PRO A:105 (hydrophobic, 3.98) ILE B:141 (hydrophobic, 3.65) TYR B:169 (halogen bond, 3.29)

## 4 Discussion

The present study integrated human peripheral blood transcriptomic reanalysis, mouse behavioral assessment, whole-brain RNA-seq, RT-qPCR, and molecular docking to investigate inhibitory/synaptic and neuroinflammatory mechanisms associated with diabetic neuropathic pain (DNP) and baclofen treatment. The major findings are as follows. First, peripheral blood transcriptomic reanalysis ofthe DPN/DNP-related human dataset revealed enhanced immune-inflammatory activation and reduced synaptic/GABAergic-related pathway activity. Second, exploratory whole-brain RNA-seq in DNP mice showed suppression of GABAergic/inhibitory and synaptic signatures, accompanied by activation of neuroinflammation/NF- *κ*B and glial-related pathways. Third, systemic baclofen treatment significantly alleviated mechanical allodynia and thermal hyperalgesia, partially improved neuromuscular performance, and did not impair motor coordination under the present dosing regimen. Fourth, baclofen was associated with partial and directional modulation of DNP-related brain transcriptional alterations, including a shift toward restoration of inhibitory/synaptic signatures and attenuation of neuroinflammatory pathway activity. Finally, baclofen increased brain *Gabra1* mRNA expression and showed preferential predicted binding to the GABA_B_ receptor in molecular docking analysis. Together, these findings support a multi-level model in which DNP involves both inflammatory activation and impaired inhibitory/synaptic transcriptional programs, while baclofen may exert therapeutic effects through GABA_B_ receptor engagement and partial modulation of these disease-associated molecular alterations.

DNP remains a clinically challenging complication of diabetes. Recent epidemiological and clinical studies have emphasized the increasing global burden of diabetes and diabetic peripheral neuropathy, as well as the substantial impact of painful neuropathy on function, quality of life, and healthcare utilization (Sun et al., 2022; Feldman et al., 2019; Elafros et al., 2022; Savelieff et al., 2025). Current pharmacological management of painful DPN remains largely symptomatic. Therapeutic reviews and the OPTION-DM trial indicate that commonly used agents, including amitriptyline, duloxetine, pregabalin, and gabapentin, provide clinically meaningful relief only in a subset of patients, and many patients require sequential or combination treatment (Mallick-Searle and Adler, 2024; Tesfaye et al., 2022). These limitations highlight the need to better define disease-associated molecular programs and identify mechanism-based therapeutic strategies. In this context, our study focused on two convergent pathological dimensions of DNP: impaired inhibitory/synaptic signaling and immune-inflammatory activation.

The human peripheral blood analysis revealed a reciprocal transcriptomic pattern characterized by increased immune-inflammatory signatures and reduced synaptic/GABAergic-related pathway activity in the DPN/DNP-related human dataset. These findings are consistent with the broader concept that systemic inflammation contributes to diabetic neuropathy and neuropathic pain (Feldman et al., 2019; Elafros et al., 2022). However, because the human dataset was derived from peripheral blood, these results should not be interpreted as direct evidence of central nervous system disinhibition. Rather, they suggest that DNP is accompanied by accessible systemic transcriptomic correlates involving immune activation and reduced inhibitory/synaptic-related signatures. This interpretation is supported by evidence that GABA is not restricted to neuronal signaling; human peripheral blood mononuclear cells and CD4^+^ T cells express GABA-related signaling components, and GABA can suppress inflammatory cytokine release from these immune cells (Bhandage et al., 2018). Therefore, the reduced GABAergic-related signatures observed in peripheral blood may reflect systemic impairment of immune-inhibitory regulatory programs rather than simply mirroring central neuronal deficits.

This peripheral blood result also shows conceptual overlap with tissue-level transcriptomic studies in human diabetic neuropathy. Hall et al. analyzed human dorsal root ganglion sensory neurons from individuals with painful diabetic neuropathy and reported inflammation-associated transcriptional activation together with reduced neuronal-related gene expression (Hall et al., 2022). Although their study examined disease-relevant sensory neurons whereas our human analysis focused on peripheral blood, both lines of evidence point toward a DNP-associated molecular pattern involving inflammatory activation and attenuation of neuronal or synaptic programs. The distinction is important: dorsal root ganglion tissue provides more direct mechanistic insight into sensory neuron pathology, whereas peripheral blood provides a clinically accessible systemic readout. Thus, our peripheral blood findings should be viewed as complementary to, rather than a substitute for, nervous-system tissue transcriptomics.

To further address whether central transcriptional alterations occur in DNP, we performed exploratory whole-brain RNA-seq in control, DNP, and baclofen-treated DNP mice. DNP mice showed significant negative enrichment of GABAergic signaling and inhibitory synapse signatures, together with positive enrichment of neuroinflammation/NF-*κ*B and glial activation-related signatures. These results align with previous work implicating impaired inhibitory regulation in neuropathic pain. Li et al. summarized the etiological contribution of GABAergic plasticity to neuropathic pain (Li et al., 2019), while Marshall et al. demonstrated spinal disinhibition in both experimental and clinical painful diabetic neuropathy (Marshall et al., 2017). Lee-Kubli et al. further proposed as a biomarker of spinal disinhibition in painful diabetic neuropathy (Lee-Kubli et al., 2018). Compared with these electrophysiological and biomarker-focused studies, our findings add a transcriptomic layer, showing that inhibitory/synaptic pathway suppression is also detectable at the whole-brain transcriptional level in DNP-like mice.

Recent circuit-level studies have further refined the central mechanisms of DNP. Chen et al. reported distinct roles of astrocytes and GABAergic neurons in the paraventricular thalamic nucleus in modulating diabetic neuropathic pain (Chen et al., 2025). Their work provides region- and cell-type-specific evidence that supraspinal glial and GABAergic neuronal circuits participate in DNP pathogenesis. Our whole-brain RNA-seq offers a broader but less spatially resolved view of central transcriptional changes. These two approaches are complementary: circuit-level studies identify specific neural substrates, whereas whole-brain transcriptomics can reveal coordinated pathway-level alterations across the brain. The consistency between their circuit-level findings and our pathway-level results supports the idea that both glial activation and GABAergic dysfunction contribute to DNP. Nevertheless, our whole-brain design cannot determine which brain regions or cell populations contributed most strongly to the observed transcriptional signatures.

Neuroinflammation emerged as another prominent feature of our analysis. In the mouse brain RNA-seq data, neuroinflammation/NF-*κ*B and glial activation-related signatures were positively enriched in DNP mice. This is consistent with prior evidence implicating Toll-like receptor signaling, NF-*κ*B activation, glial inflammatory cascades, and microglial reactivity in DNP (Liu et al., 2018; Wang et al., 2024). Recent work has emphasized that microglia may promote and maintain diabetic neuropathic pain through inflammatory mediator release, receptor-mediated activation, and modulation of neuronal excitability (Wang et al., 2024). In our study, the neuroinflammation/NF-*κ*B signature was increased in DNP mouse brain and shifted downward after baclofen treatment, suggesting that inflammatory pathway modulation may contribute to baclofen-associated analgesic effects. Thus, our results support a model in which inflammatory activation and inhibitory/synaptic suppression coexist and may interact to promote central sensitization and persistent pain.

Baclofen significantly alleviated mechanical allodynia and thermal hyperalgesia in DNP mice. These results are consistent with previous preclinical studies showing that GABA_B_ receptor activation can reduce diabetic neuropathic pain. Liu et al. reported that intrathecal baclofen inhibited p-CREB and NR2B expression in the spinal dorsal horn of DNP rats (Liu et al., 2014), and another study demonstrated that GABA_B_ receptor activation suppressed TLR4/MyD88/NF-*κ*B signaling in the spinal dorsal horn (Liu et al., 2018). Our study differs from these prior spinal cord-focused studies in three ways. First, we used systemic baclofen administration rather than intrathecal delivery. Second, we assessed pain behavior together with neuromuscular function and motor coordination. Third, we incorporated whole-brain RNA-seq to examine treatment-associated transcriptional modulation. Therefore, our data extend previous findings by suggesting that baclofen may be associated with broader central transcriptional changes involving both inhibitory/synaptic and inflammatory pathways.

A key consideration in interpreting baclofen’s behavioral effects is the possibility of nonspecific motor suppression. GABA_B_ receptor agonists can produce sedation, muscle relaxation, or motor deficits at excessive doses, which may confound pain behavioral readouts. GABA_B_ receptors are metabotropic receptors that regulate neuronal excitability through presynaptic and postsynaptic mechanisms, and their pharmacological activation requires careful behavioral interpretation (Bettler and Tiao, 2006). In our study, baclofen improved pain thresholds and partially restored grip strength without reducing rotarod performance, suggesting that the observed analgesic effects were unlikely to be caused by nonspecific motor impairment. The partial improvement in grip strength may reflect reduced pain burden and improved functional performance, although direct effects on neuromuscular physiology cannot be excluded.

At the transcriptomic level, baclofen did not globally normalize the DNP brain transcriptome. Instead, it partially and directionally modulated selected DNP-associated pathways. In the BAC versus DNP comparison, GABAergic signaling and inhibitory synapse signatures shifted toward positive enrichment, while neuroinflammation/NF-*κ*B shifted downward. The reversal quadrant analysis further showed that a subset of DNP-regulated genes changed in the opposite direction after baclofen treatment. This pattern is biologically important because it supports selective transcriptional modulation rather than complete disease reversal. Therefore, baclofen should not be described as globally restoring the DNP transcriptome. A more appropriate interpretation is that baclofen partially counteracts selected inhibitory/synaptic and inflammatory transcriptional abnormalities associated with DNP.

The qPCR result showing increased *Gabra1* expression after baclofen treatment provides additional support for baclofen-associated modulation of inhibitory signaling. *Gabra1* encodes the *α*1 subunit of the GABA_A_ receptor and contributes to fast inhibitory neurotransmission. Reduced *Gabra1* expression in DNP mice is consistent with impaired inhibitory tone, whereas its increase after baclofen treatment suggests that GABA_B_ receptor activation may be associated with downstream transcriptional changes involving GABA_A_ receptor-related components. However, this result should be interpreted cautiously. The present data do not prove direct receptor cross-talk between GABA_B_ activation and GABA_A_ receptor upregulation. Instead, *Gabra1* restoration should be viewed as a molecular correlate of improved inhibitory pathway status. Protein-level validation, receptor localization, electrophysiological recordings, and pharmacological blockade experiments will be required to determine whether increased *Gabra1* mRNA translates into enhanced functional GABA_A_ receptor-mediated inhibition.

The potential interaction between baclofen and inflammatory signaling is also supported by studies outside DNP. Crowley et al. reported that baclofen modulates *TLR3/TLR4* inflammatory signaling in glial and immune cells, suggesting that GABA_B_ receptor activation may influence innate immune responses (Crowley et al., 2015). Bhandage et al. further showed that GABA suppresses cytokine release from human PBMCs and CD4^+^ T cells (Bhandage et al., 2018). These findings provide a mechanistic context for our observation that baclofen-treated DNP mice showed a downward shift in neuroinflammation/NF-*κ*B-related pathway activity. Although our data do not establish a direct causal link between baclofen and immune-cell signaling in the brain, they suggest that modulation of inflammatory transcriptional programs may contribute to the therapeutic effects of GABA_B_ receptor activation.

Molecular docking analysis provided structural support for the preferential interaction of baclofen with the GABA_B_ receptor. Baclofen showed a more favorable predicted binding energy with GABA_B_ than with GABA_A_ and formed key interactions within the GABA_B_ Venus Flytrap domain, including a predicted salt bridge with Arg162. These findings are consistent with the known pharmacological profile of baclofen as a selective GABA_B_ receptor agonist (Bettler and Tiao, 2006). In addition, Hleihil et al. reported that sustained baclofen-induced activation of GABA_B_ receptors after cerebral ischemia restored receptor expression and function and limited neuronal loss (Hleihil et al., 2021). Although this study was performed in a cerebral ischemia model rather than DNP, it supports the broader concept that GABA_B_ receptor activation may influence receptor expression and neuronal function under pathological conditions. Nevertheless, docking results should be interpreted as supportive rather than definitive evidence. Docking predicts possible binding poses and relative interaction energies, but it does not measure receptor activation, binding kinetics, or downstream signaling. Therefore, the docking analysis complements the behavioral, transcriptomic, and qPCR results by supporting target plausibility, but it cannot independently establish the mechanism of baclofen’s analgesic effect.

### 4.1 Limitations and future perspectives

Several limitations should be acknowledged. First, the human transcriptomic analysis was based on peripheral blood, which provides accessible systemic information but cannot directly demonstrate central nervous system mechanisms. In addition, the human GSE95849 dataset was originally annotated as diabetic peripheral neuropathy rather than strictly painful DNP; therefore, the human peripheral blood analysis should be interpreted as DPN/DNP-related supportive evidence rather than direct transcriptomic evidence from clinically confirmed painful DNP patients. Although the mouse whole-brain RNA-seq analysis provided central tissue-level support, it did not resolve region-specific or cell-type-specific changes. Future studies using spinal dorsal horn, dorsal root ganglia, thalamic nuclei, somatosensory cortex, or limbic pain-processing regions would help define the anatomical sources of the observed transcriptional alterations. Single-nucleus RNA-seq, spatial transcriptomics, or cell-type-specific validation may further clarify the contributions of neurons, astrocytes, microglia, and other cell populations.

Second, the RNA-seq analysis included a limited number of biological replicates and should be considered exploratory. Third, although baclofen increased brain *Gabra1* mRNA expression, protein-level and functional validation remain necessary. Western blotting, immunofluorescence, receptor localization analysis, and electrophysiological recordings would be useful to determine whether *Gabra1* transcriptional restoration translates into enhanced GABA_A_ receptor-mediated inhibition. Fourth, only male mice and a short treatment window were examined; future studies should evaluate sex-specific responses, dose-response relationships, long-term efficacy, and potential tolerance. Finally, molecular docking provides predictive structural evidence but cannot replace experimental binding or receptor-activation assays.

## 5 Conclusion

This study demonstrates that DNP is associated with convergent peripheral and central transcriptomic alterations involving enhanced immune-inflammatory activation and reduced inhibitory/synaptic pathway activity. Human peripheral blood transcriptomic reanalysis revealed systemic inflammatory activation and reduced synaptic/GABAergic-related signatures, while mouse whole-brain RNA-seq further supported central suppression of GABAergic/inhibitory pathways and activation of neuroinflammation-related programs. Baclofen significantly alleviated mechanical and thermal hypersensitivity and was associated with partial, directional modulation of DNP-related brain transcriptional changes. Together with increased brain *Gabra1* expression and preferential predicted binding to the GABA_B_ receptor, these findings suggest that baclofen may attenuate DNP by engaging GABA_B_ receptor signaling and partially rebalancing inhibitory/synaptic and inflammatory transcriptional programs.

## Data Availability

The human peripheral blood transcriptomic data analyzed in this study are publicly available in the NCBI Gene Expression Omnibus (GEO) under accession number GSE95849. The mouse whole-brain RNA-sequencing data generated in this study have been deposited in the NCBI Gene Expression Omnibus (GEO) under accession number GSE343056.
